# Clinical and Rehabilitative Management of Retinitis Pigmentosa: Up-to-Date

**DOI:** 10.2174/138920211795860125

**Published:** 2011-06

**Authors:** Francesco Parmeggiani, Giovanni Sato, Katia De Nadai, Mario R Romano, Andrea Binotto, Ciro Costagliola

**Affiliations:** 1 Department of Ophthalmology, University of Ferrara, Ferrara, Italy; 2 Eye Clinic, S. Antonio Hospital, ULSS 16 Padova, Padova, Italy; 3 Center for Retinitis Pigmentosa of Veneto Region, Camposampiero Hospital, ULSS 15 Alta Padovana, Camposampiero, Italy; 4 Department of Health Sciences, University of Molise, Campobasso, Italy

**Keywords:** Inherited retinal dystrophies, retinitis pigmentosa, multidisciplinary management, clinical governance, prevention, therapy, rehabilitation.

## Abstract

The term retinitis pigmentosa (RP) indicates a heterogeneous group of genetic rare ocular diseases in which either rods or cones are prevalently damaged. RP represents the most common hereditary cause of blindness in people from 20 to 60 years old. In general, the different RP forms consist of progressive photo-receptorial neuro-degenerations, which are characterized by variable visual disabilities and considerable socio-sanitary burden. Sometimes, RP patients do not become visually impaired or legally blind until their 40-50 years of age and/or maintain a quite acceptable sight for all their life. Other individuals with RP become completely blind very early or in middle childhood. Although there is no treatment that can effectively cure RP, in some case-series the disease’s progression seems to be reducible by specific preventive approaches. In the most part of RP patients, the quality of vision can be considerably increased by means of nanometer-controlled filters. In the present review, the main aspects of the routine clinical and rehabilitative managements for RP patients are described, particularly focusing on the importance of specific referral Centers to practice a real multidisciplinary governance of these dramatic diseases.

## INTRODUCTION

Inherited retinal dystrophies are a heterogeneous group of rare diseases affecting the posterior segment of the eye [1-6]. In the course of routine clinical practice, the different forms of retinitis pigmentosa (RP) are the most frequently diagnosed heredo-dystrophic pathologies of the retina, being neurodegenerative disorders of the *tapetum* , which represents a layer composed by perennial cells named retinal photoreceptors, i.e. rods and cones. The definition “tapeto-retinal degeneration” has been firstly utilized by Leber in 1916, describing an ocular disease that Donders in 1857 defined as “retinitis pigmentosa”. Although this latter expression is not properly corrected, because the inflammation is not the main process in these eye disorders, retinitis pigmentosa is currently worldwide used. In the different ethnic groups, RP prevalence is variable reported in 1 case for each 3000-5000 individuals [7-23], even if among particular populations the disease’s occurrence seems to be very higher [24, 25]. The different clinical RP-patterns are generally progressive and bilateral. Each of these phenotypes is hereditable in line with all the typologies of the Mendelian inheritance. The various typologies of RP represent very complex eye diseases from both genotypic and phenotypic point of view. According to the clinical manifestations, two main groups of RP are schematically distinguishable: i. typical RP or rod-cone dystrophy (RCD) (about 80-90% of the total cases), in which the rods are predominantly damaged; ii. atypical RP or cone-rod dystrophy (CRD) (about 10-20% of the total cases), in which the cones are primarily injured. In the most part of patients with RCD or CRD (approximately 85% of cases), these ocular degenerative disorders are considerable as isolated diseases, i.e. non-syndromic RPs. However, many systemic disorders can be associated with various types of pigmentary retinopathies. In the large majority of patients affected by syndromic inherited retinal dystrophies, the clinical manifestation of their ocular involvement consists of a typical form of RP. These syndromic RPs approximately represent the 15% of the total cases suffering from tapeto-retinal degeneration. Usher syndrome is the most frequent syndromic disorder, in which typical RP is associated with neuro-sensory deafness. About 14% of all RP cases are, in fact, Usher syndrome [4, 8, 13, 15, 16]. The deafness, normally congenital and stable, may be severe (type 1) or moderate (type 2); in other cases, it occurs during the first decade and progressively worsens (type 3).

As already mentioned, the RCD is characterized by a progressive neuro-degenerative damage mostly affecting rods, which are the retinal photoreceptors able to ensure both the nocturnal and the peripheral visions. In patients affected by early-stage RCD, a decreasing of the visual abilities in the dark (nyctalopia), a slight to moderate shrinking of the visual field and/or a remarkable dazzle sensation (photophobia) are often reported. This latter symptom is commonly detectable also in patients suffering from early-stage CRD. Moreover, because the cones are prevalently injured, these individuals usually complain about noticeable alterations of both quantity and quality of their central vision. Although many patients with late-stage RCD have peripheral or total blindness and numerous patients with late-stage CRD are centrally blind, the natural history of all tapeto-retinal degenerations is often unpredictable. In fact, the final visual prognosis of each RP patient can be dependent not only on genetic factors (such as different expressivity and/or penetrance of the causative disease-gene) [4-6, 26-28] but, sometimes, also on environmental factors (such as different levels of eye-exposure to harmful light radiations) [29-36].

The aforementioned evidences, indicative of both interfamilial genotypic multiplicity and intra-familial phenotypic variability of RP, are unavoidably related to a complex and customized management of each patient with either RCD or CRD, which involves peculiar clinico-genetic, psychological and rehabilitative aspects.

## MULTIDISCIPLINARY MANAGEMENT

In Caucasian ethnic groups, the total prevalence of RP is estimable in 32.2 cases per 100 000 persons [17]. This number is relatively low in comparison with the general population, but it is of critical importance especially considering the socio-sanitary characteristics of RP, together with its insidious and/or grave consequences. In fact, the different forms of RP often represent very disabling disorders, progressively more severe already during either school- or working-age, without any definite therapeutic strategy and, in many cases, associated with significant risk of hereditary transmission. Therefore, also to avoid an attitude of renunciation-care by several RP patients, the possibility of referring to a specialized Center, able to serve a catchment’s area inhabited by at least 1.5-2.5 millions of people, seems to be an appropriate option for the clinical governance of this rare eye disease. The presence of these referral RP services is essential to ensure that multidisciplinary approach in which different professionals work together trying to give feasible solutions to patient’s requests and/or effectual responses to those queries that a person with RP usually turns to the ophthalmologist:

- what is my visual capacities compared to those of healthy population?
- what kind of visual impairment should I expect and when?
- what is the better rational treatment for my disease?
- is it possible that other disorders (such as deafness, cataract, macular edema, ocular hypertension and/or glaucoma) come out?
- in these latter cases, how will you can treat me?
- what is the risk of RP transmission in my family?
- what socio-economic benefits and welfare rights can I get?
- can you do anything to improve my quality of vision and my quality of life?

The ideal clinical and rehabilitative management of patients with ascertained or suspected RP becomes practicable when the ophthalmologist is in a position to coordinate a work-team necessarily composed by other health-care professionals, who can be alternately or synergistically exploited. This multifaceted board should include:

- ophthalmologist, the coordinator of the work-team, who achieves the standardized phenotyping of each RP patient, assessing visual functions, planning the clinical follow-up, managing preventive, rehabilitative, medico-legal and/or epidemiological aspects, also acting in cooperation with patient’s association and/or with dedicated social networks;
- geneticist, who accomplishes the genetic counseling of RP patients or families, dealing the medical genetics, indicating the possibilities of molecular diagnosis, the perspectives of gene therapy, and the pharmacogenetic aspects;
- audiologist, a specialized health-care professional, who identifies, diagnoses, monitors and treats the disorders of the auditory and vestibular system portions of the ear;
- other specialized health-care professionals (just for example, nephrologist, dermatologist and neurologist) who define, diagnose, check and treat the various systemic disorders present in patients with syndromic RP forms;
- assistant in ophthalmology or orthoptist, a vision rehabilitator who periodically works, in case supported by an optometrist or optician, to optimize the practical training of each RP user of optical and/or electronic aids, improving eye movements (conjugate and/or tracking), fixation and its maintenance especially for the reading, PC and/or TV utilizations;
- typhologic and occupational therapist, who periodically works to optimize the practical training of each RP user of typhlo-tecnical aids, and to enhance the eye-hand collaboration, movement execution (skill, speed and/or precision), ability to handle and recognize objects;
- orientation and mobility trainer, responsible for education and training to make RP patient able to be autonomous in movements both in known and unknown locations;
- psychologist, who evaluates the psychological distress of an individual suffering from RP and/or his/her family, in case providing psychological support for a social re-integration of the patient (especially at school or at work) and also acting in cooperation with the social assistants.

In the next parts of the present review, the assisting approaches dedicated to patients with RP will be focused and discussed.

## CLINICAL GOVERNANCE

The main commitments of a referral Center specialized in the clinical governance of patients affected by RP can be summarized considering, at least, these essential activities:

1. correct diagnostic classification of each RP-case and early diagnosis, employing both conventional and multifocal electroretinograms, visual field examinations, microperimetry, retinographies, retinal angiographies, and optical coherence tomography (OCT);
2. certification of ocular rare disease, epidemiologic and medico-legal evaluations, information about opportunities and limitations of social-health system;
3. anamnestic study concerning the possible inheritance, genealogic trees and risk estimation of the heredofamilial transmission;
4. appropriate and standardized phenotyping of each RP patient, accompanied by the collection of biological material aiming to DNA storage and extraction in view of the current chances of molecular diagnosis and/or the future prospects of broad spectrum DNA bio-molecular tests;
5. correct and realistic information about the current curative options for RP and their possible side effects;
6. specific and personalized protocols intended to optimize prevention, treatment and/or rehabilitation of every patients suffering from different forms of RP;
7. specific and personalized protocols intended to effectively manage the concomitant ocular disorders possibly correlated with RP, such as cataract and cystoid macular edema;
8. recommendations regarding lifestyle, such as not smoking, low-fat diet accompanied by abundant fruits and vegetables, and regular aerobic exercise;
9. general and specific recommendations concerning the assumption of drugs or herbal medications.

In particular, this latter point should be considered in the cases of ascertained or suspected iatrogenic interactions, and to possibly avoid in RP patients the following drugs: i. potential retino-toxic compounds, such as the cGMP-specific phosphodiesterase type-5 inhibitors (erectile dysfunction drugs), isotretinoin and other retinoids, anti-psychotic and anti-histaminic drugs containing phenothiazines, vigabatrin (an anti-epileptic drug), aminoquinoline (an anti-malarial drug), tamoxifen (an antagonist of the estrogen receptor), and high dosage of hydroxychloroquine (a drug used to treat or prevent malaria and, more frequently, to treat lupus and rheumatoid arthritis); ii. potential neuro-toxic compounds, such as ethambutol (an anti-mycobacterial drug), linezolid (a synthetic antibiotic), and amiodarone (an anti-arrhythmic agent); iii. drugs associated with potential risks of acute or intermittent angle closure glaucoma in susceptible individuals, such as tricyclic antidepressants and other agents with anti-cholinergic properties, serotonin-norepinephrine reuptake inhibitors and selective serotonin reuptake inhibitors (the most commonly used antidepressant drugs), adrenergic agents, and certain beta-2 adrenergic agonists [37-41]. In RP patients who need one or more of the aforementioned drugs, they should always be used under careful ophthalmologic supervision.

## CONVENTIONAL CURATIVE STRATEGIES

In the course of the routine clinical practice of a referral Centers dedicated to the clinical management of RP, the most frequent patient’s query concerns the therapeutic possibilities to block or reduce the progression of the degenerative retinal disorders. Although many curative attempts have been hitherto carried out, currently there is no definitive treatment for RP [42-44]. In fact, all these therapeutic approaches have not proved effective when subjected to critical review according to the criteria of the evidence-based medicine (EBM). For this reason, it is not possible to define any shared interventional guideline for the care of RP patients. However, the ophthalmologist of a referral RP Center can rationally recommend several strategies aimed to reduce the phenotypic severity of these neurodegenerative rare diseases of the ocular posterior segment. In general, these therapeutic possibilities, which may be labeled as “conventional treatments”, should not induce false expectations in patients and, thus, each of them must be proposed in a very rigorous manner, clearly detailing that:

- no significant chance of visual recuperation is envisage to patients;
- the most optimistic hope is represented by the stabilization or by a slowdown of the detrimental trend of the disease’s progression;
- both the disease’s progression and the final visual prognosis are mainly related to several genetic factors, from which the individual expressivity of each disease-gene depends and against which nothing is actually effective at present.

In patients with a rare disease characterized by absence of EBM-supported treatments and high risk of severe disability, such as RP, the prescriptive attitude must be unavoidably based just on clinical case-series data and/or on experimental evidences. Starting from this deontological point of view, the decision-making therapy should be performed especially considering the risk/benefit ratio and, to minor extent, the cost/benefit ratio of each treatment. In particular, some curative strategies for RP, operating with synergistic mechanism of action, are proposable to temptatively downgrade the clinical worsening of this chronic eye disease in the long-term period:

- nanometer-controlled filtering lenses (light protection or anti-phototoxic medical device) [29-36];
- supplementation with vitamin A palmitate (associated or not with the intake of docosahexaenoic acid or lutein) [45-52];
- other nutritional supplements and off-label drugs, i.e. lutein supplementation [53, 54], nilvadipine [55-57], and 9-cis-beta-carotene [58].

### Nanometer-Controlled Filtering Lenses

Blue light-filtering and ultraviolet light-filtering lenses reduce the phototoxic effects on retina. In particular, the sunlight contains a wide range of wavelengths (λ), part of which is harmful for the ocular structures, i.e. ultraviolet radiation (λ = 200-400 nm), high-energy violet (λ = 400-440 nm) and blue (λ = 440-500 nm) lights. The retinal photo-toxicity of these radiations has been demonstrated in experimental models of RP. Moreover, several data from animal studies indicate that some pigmentary retinopathies are peculiarly susceptible to light damage [30-32, 34, 35]. The continuative use of one or more types of nanometer-controlled filters can significantly reduce both ultraviolet and blue lights negative effects at the level of vitreoretinal tissues suffering from RP. 

In the last years, these anti-phototoxic medical devices are available in numerous colorations, each of which is able to partially or totally block harmful wavelengths of the light. In particular, patients with RP are recommended to wear dark nanometer-controlled glasses outdoors. The employment of these amber spectacles should be useful to counteract the damages of ultraviolet rays and visible wavelengths up to about 511 nm or 527 nm. Ideally, to protect from the sunlight the best option is represented by the utilization of lenses blocking ultraviolet rays and radiations up to approximately 550 nm to filter blue-violet light. On the other hand, patients with RP can be also advised to wear clear nanometer-controlled spectacles both outdoors in cloudy days and indoors to diminish dazzle during PC/TV monitors use or under the illumination of halogen lamps. Although habitually yellow, orange or red filtering lenses are prescribed to minimize photophobia, because of less chromatic aberrations numerous RP patients experience a better tolerance and compliance by the use of filters also containing significant part of brown, mixed with the basic yellow, orange or red tints.

In addition, nanometer-controlled filters have an important role in rehabilitative management of RP patients [59, 60]. In fact, these devices optimize the quality of vision in the majority of individuals with RCD or CRD, increasing contrast sensitivity and decreasing glare. For the aforementioned reasons, many patients affected by RP decide to employ at least two pair of specific filtering glasses with different levels of light-absorption and light-protection basing on the ambient brightness conditions in which they are. Nanometer-controlled filters are available as polarized and non-polarized lenses and, of course, they can correct eventual refractive errors of the patient (myopia, hypermetropia and/or astigmatism). Finally, eyeshade and lateral protection can facilitate the protection against dazzling side-coming light rays. In theory, there is the possibility to utilize photo-chromic filters, even though they cannot be able to ensure the same degree of protection obtainable with the photo-static ones.

The patients with RP have usually need of continuative filters exploitation after the carrying out of cataract surgery, even if filtering intraocular lenses are implanted. Nanometer-controlled sunglasses provide about 50% more ultraviolet/blue photo-protection than either violet or blue blocking intraocular lenses [61], nevertheless these specific surgical devices should be systematically used in all RP candidates to cataract extraction procedure. Optimal risk/benefit and cost/benefit ratios characterize the employment of nanometer-controlled filters, despite they are associated with alterations of colors perception. The comprehensive consideration of the above-described aspects makes desirable that Health Systems provide for the reimbursement of all the nanometer-controlled lenses required by each patient with early- or late-stage RP.

### Vitamin A Palmitate

The vitamin A supplementation may be notionally able to protect the photoreceptors by trophic and antioxidant effects. In the course of the last 15-18 years, Berson and co-workers have periodically reported that vitamin A palmitate in long-term doses of 15,000 IU per day slowed down the photo-receptorial functional damages studying several clusters of patients affected by heterogeneous forms of typical RP [45-51]. At present, the ophthalmologists continue to debate the conclusions of these investigations, particularly questioning about “How strong is the evidence that nutritional supplements slow the progression of retinitis pigmentosa?” [52]. Although no definitive consensus has been reached on the usefulness of vitamin A, this treatment seems to be more efficient in cases of RP caused by mutations in RHO1 gene (see the study design of the clinical trial NCT00065455 in http://clinicaltrials.gov/). If a prolonged high-dosage supplementation of vitamin A palmitate is proposed, periodical blood test must be scheduled to monitor the levels of serum retinol (normal < 3.49 μmol/l, i.e. < 1 mg/l) and triglyceridemia (normal < 2.13 mmol/l, i.e. < 0.19 g/l), at least together with the check of the main liver enzymes (aspartate aminotransferase, alanine aminotransferase, and alkaline phosphatase) because the iatrogenic vitamin A accumulation prevalently occurs in this organ. In case of ascertained or suspected signs of hepato-toxicity the treatment must be discontinued. Moreover, considering that vitamin A should not be given to patients with RP caused by mutations in ABCA4 gene, specific genotypic analyses might be temptatively indicated before starting this supplementation to better define exclusion criteria [4, 44, 62]. Likewise, starting from a phenotypic point of view, also the presence of hepatic disorders, potentially linked to excessive risk of drug-toxicity, should be considered to rule out some RP patients from treatment with high-dosage vitamin A.

In a subsequent study, Berson and co-workers have verified the effect of docosahexaenoic acid supplementation at 1200 mg/day in addition to vitamin A, indicating that the pathologic course of RP was downgraded by docosahexaenoic acid, but this positive outcome did not persist in the long-term period. Moreover, RP patients taking vitamin A palmitate, but not docosahexaenoic acid, benefited from an omega-3 rich diet (equivalent to eating salmon, tuna, mackerel, herring and/or sardines, once to two times a week) [49, 50]. Recently, the same group of researchers has conducted a 4-year clinical trial on RP patients taking vitamin A randomized to either placebo or lutein (12 mg/day) regimen, concluding that this latter combined treatment is able to better counteract the visual field decline [51].

Without identification of the RP disease-gene, as well as without preliminary tests aimed to diagnose eventual hepatopathies, the risk/benefit ratio of high-dosage supplementation of vitamin A palmitate is not exactly definable. Hence, this therapeutic strategy should be utilized under a careful supervision of: i. visual functions (to promptly identify unexpected and/or potentially iatrogenic retinal deteriorations); ii. possible systemic side effects.

### Nutritional Supplements and Off-Label Drugs

Other therapeutic approaches should be considered during the personalized clinical management of each RP patient, in particular:

- supplementation with lutein, a xanthophyll representing one of numerous naturally-occurring carotenoids, which, at 12 mg/day or more, may increase its physiologic effects in keeping the eyes safe from both oxidative stress and high-energy photons of blue light [53, 54];
- nilvadipine, a calcium-channel blocker drug marketed for the treatment of high blood pressure and also for cerebral vascular disorders, which, at 4 mg/day, seems to be able to retard progression of central visual field defects in RP patients hypothetically through its neuroprotective anti-amyloid actions [55-57];
- exclusively in patients with fundus albipunctatus, a quite benign form of inherited RCD caused by a mutation in the gene encoding 11-cis-retinol dehydrogenase and mainly characterized by congenital stationary night blindness, the oral treatment with 9-cis-beta-carotene has been recently reported to recover the photo-receptorial changes secondary to this retinal dystrophy – the same therapy might be evaluated in other types of human RP with similar gene-related pathogenetic mechanisms [58].

Considering the favorable safety profile of all the above-mentioned medical therapies, both their risk/benefit and cost/benefit ratios appear to be acceptable. Similarly, also rasagiline, a selective inhibitor of monoamine oxidase type B utilized as anti-Parkinson drug, may be prescribed, even if its anti-apoptotic action has been documented just in animal model of RP [63].

Finally, it could be eventually advisable the intake of various and/or combined nutritional supplements that potentially act as anti-oxidants, immuno-modulators, microcirculation adjuvants and/or photo-protectors. Although no supporting controlled datum exists, they notionally benefit the retina in which there can be a very high level of free radicals, as it happens in RP patients.

## INTERVENTIONAL STRATEGIES FOR OCULAR COMPLICATIONS

In the course of the clinical practice of a referral Center for RP patients, it is necessary to carry out the most appropriate diagnostic and therapeutic approaches for the management of some specific eye disorders associated with RP. Particularly, the most frequent complications are cataract and macular edema.

### Cataract

In the majority of patients affected by RP, the typical form of crystalline lens opacity is represented by a posterior central sub-capsular cataract with a clear nucleus, which is variably present at early or mid stage of the disease’s evolution. Although this cataract is not widespread, its central location can partially blur the central and peri-central vision. However, the surgical procedure (i.e. phacoemulsification with implantation of intraocular lens) is not recommendable at initial cataract stages, especially for the risk to generate or upgrade photophobia. Once RP patient was correctly informed on the risk/benefit ratio of cataract surgery in his/her specific case, as well as considering the physiologic photo-protection related to the presence of crystalline lens, this wait-attitude appears to be rational and, habitually, very appreciated by patients. When the surgical procedure becomes necessary (usually at least after the development of a moderate senile cataract component), it should be proposed with special attention to: i. detailed information to patient (both positive and negative aspects); ii. opportunity to plan the employment of filtering intraocular lens [64, 65]; iii. scheduling specific diagnostic investigations before and after surgical procedure (i.e. optical coherence tomography of the macular area) [66]; iv. option to prescribe pre- and post-operative regimens to reduce the risk of occurrence or aggravation of post-surgical cystoid macular edema (topical and/or oral non-steroidal anti-inflammatory drugs, eventually together with topical and/or oral carbonic anhydrase inhibitors) [67-70].

### Macular Edema

In patients suffering from RP, macular edema frequently occurs, causing a variable decrease in the visual acuity and/or contrast sensitivity. It is typically characterized by the presence of cystoid intra-retinal alterations, and its pathogenesis is mainly related to the degenerative changes affecting the vitreoretinal structures of the posterior ocular segment. The cystoid macular edema occurring in RP patients may be also caused by tractional changes in the vitreoretinal limiting membrane [71], as well as by inflammatory conditions and/or events (such as eventual post-surgical disorders). Although the cystoid macular edema in RP patients is most often chronic, several pathologic forms may be successfully treated with carbonic anhydrase inhibitors such as acetazolamide sodium at a daily dose of 500 mg or less [72-74]. The continuative utilization of this drug should be performed under strict medical supervision. In some case-series, also the topical administration of dorzolamide is efficient in downgrading cystoid macular edema [75-78]. Finally, either intravitreal or sub-Tenon posterior triamcinolone acetonide injection could be employed for select cases of cystoid macular edema in RP patients but its efficacy seems to be limited over time [79-83].

## CRITICAL REVIEW OF OTHER INTERVENTIONAL ATTEMPTS

In the last one-hundred years, more than fifty very different treatments for RP have been proposed and evaluated. Unfortunately, none of them has turned out to be effective according to the EBM criteria. These approaches are briefly, even if incompletely, condensable as follows: i. autologus or heterologus retro-choroidal grafts; ii. intravitreal, peribulbar, retrobulbar and/or subconjunctival injections with various drugs, herbal medications, vitamins, enzymes, antioxidants, mineral salts, L-DOPA, retinal lipoid extracts, vasodilators, platelet inhibitors, carbonic anhydrase inhibitors, immuno-modulatory agents; iii. laser therapy, magnetic therapy, ultrasound therapy, acupuncture, electrical stimulation, neuro-sensorial photo-stimulation through photoreceptor biofeedback and so on. Considering both risk/benefit and cost/benefit ratios, all the above-listed therapeutic attempts are currently not recommended by the Health Systems of United States, Canada, Europe and Australia, as well as in other many Nations. In particular, an interventional strategy had become quite common among patients with RP during the last three decades, i.e. the so-called “Cuban therapy”. This intervention comprises: i. surgical approach, wrongly labeled as “revitalizing”, which consists in the insertion of autologus orbital adipo-vascular tissue into a 180-degree sclerochoroidal pocket at the temporal side of the eyeball; ii. periodical sessions of intravenous ozone-therapy; iii. periodical cranium-orbital electro-stimulating treatments; iv. oral administration of anti-platelets, antioxidants and immuno-modulators. Performing this approach and comparing it with the natural history of RP in 195 patients, Peláez and co-workers have reported rather encouraging results, especially in the early stages of the disease [84-86]. However, these data have been never validated and, at present, the “Cuban therapy” is not performed and not advisable [87-90], as well as other similar surgical strategies: i. the so-called “Russian therapy” consisting in the retro-choroidal grafts of powdered extracts of heterologus biological material (named “alloplant”) [91, 92]; ii. the so-called “Cuban-modified therapy” consisting in the insertion of autologus orbital adipo-vascular tissue into a 20-degree temporal sclerochoroidal pocket.

Finally, another interventional strategy, characterized by both far-and-wide debated mechanism of action and criticizable risk/benefit ratio, is still infrequently applied to RP patients, i.e. the hyperbaric oxygen therapy. In few series of patients with RP, this long-term treatment has been supposedly able to stabilize the disease’s changes measured with perimetric and/or electroretinographic exams [93, 94]. These outcomes have indicated that hyper-oxygenated regimen could be related to a better chance of slowing RP progression in respect of the high-doses treatment with vitamin A [94] but, at present, it is generally not recommended, as well as any other hyper-oxygenation tissular strategy, such as intravenous ozone therapy. In fact, an increase of hyper-oxidant risks has been postulated, mainly owing to the release of oxygen free radicals and to the aberrant overloading of catabolic products harmful to retina [95-97]. Both these mechanisms are potentially related to a final worsening of the visual prognosis in RP patients.

## REHABILITATIVE MANAGEMENT

Several patients with RP gradually experienced different typologies of important visual impairment and disability. Specific rehabilitation trainings are usually very helpful and, thus, they should be timely programmed. In the severe cases of either RP/RCD (prevalently associated with peripheral visual loss) or RP/CRD (prevalently associated with central visual loss), low-vision aids are frequently useful: hand-held or stand magnifiers, half-eye base-in prism lenses, telescopes, hand-held or stand electronic devices and other equipments (above all in RP/CRD cases), possibly together with orientation and mobility training (above all in RP/RCD cases) [59, 60, 98-102]. Although the reading performance of most patients with RP/RCD is impaired not only for alterations of contrast sensitivity and visual acuity but also for visual field constriction [103], the utilization of electronic vision enhancement systems may be advisable in several individuals, especially the young ones. The completion of visual rehabilitation can improve daily-life activities and a high level of patient’s satisfaction is often reported. In all individuals affected by RP, a particular emphasis should be put on nanometer-controlled filtering lenses, which are commonly able to minimize photophobia outstandingly increasing the quality of vision [59, 60, 98]. The rehabilitation processes are aimed to maintain patient’s independence at home and in the community; they involve an accurate assessment of the sight functions, followed by peculiar exercises with optical and/or electronic vision aids.

In the most part of RP patients suffering from low-vision or blindness, the rehabilitative management is complex especially because the person to be rehabilitated is in school or working age. In fact, these individuals are often severely disabled or legally blind by the end of the second, third or fourth decade of life. For that reason, it is important that their education focuses on an adapted professional occupation (telephone operator, teaching, computer based activities, physiotherapist). Moreover, psychological support is often necessary in the course of several landmarks regarding a neurodegenerative inherited disease, such as RP: announcement, information about the procreative risks, occurrence of moving difficulties, loss of reading and/or other visual troubles. This care can be provided by either professionals or supportive patients associations. The disabled persons with RP should be also oriented to Institutions that help them to rehabilitate (short- and medium-stay stages) and/or to obtain new professional skills. Independently from the causative tapeto-retinal disorder, important benefits are often obtainable in RP patients suffering from low-vision or blindness, respectively, by means of rehabilitative or typhlo-tecnical devices, enabling them to take part in several occupational/social activities and to greatly improve their quality of life. The final aim is to optimize the remaining visual capacities or the other perceptive capacities, so that an individual can, at least partially, continue to do routine tasks and job activities, reaching meaningful personal aspirations.

## CONCLUSION

At present, the clinical management of the different RP forms should be carried out optimizing that multidisciplinary approach essential to meet all the complex care needs of each patient. Ideally, this optimization is achievable through the continuous activities of a consolidated work-team, coordinated by an ophthalmologist responsible of specific referral Centers for RP. In a shared view of collaborative network, each RP Center should be able to agree with the others about the main clinical guidelines standardizing, at least, a comprehensive phenotyping protocol, a minimal therapeutic planning and some psycho-rehabilitative approaches. Therefore, both the chief ophthalmologist and the other professional employees of a specific health-care Center for an ocular rare disease, such as RP, should be expressly trained to handle the multifaceted aspects of each case. Despite various curative strategies are available, or becoming potentially available, to restore or stabilize vision loss caused by RPs, currently the most part of these therapies do not be definitely validated in humans. Comprehensively taking into account several scientific, medical, deontological and psychological aspects, the lack of any effective treatment for RP makes difficult a categorical choice between different prescriptive attitudes toward this very severe pathology; in fact, the application of either EBM or complementary and alternative medicine criteria [104] may be inadequate in the context of RP decision-making. The emotional impact to researchers, clinicians, patients, and families from the recent results in molecular diagnosis, gene therapy and other pioneering treatments is very evident [6, 105-130]. Certainly, the above-cited considerations must not lead to excessive patient’s expectations and, above all, must not divert attention from that should be recommended during the routine clinical practice. However, several realistic chances of bridging the gap from RP lab to RP patients are getting closer. In the near future, an extensive application of these opportunities appears to be feasible only if a reinforced educational attitude will increase the number of ophthalmologists and geneticists able to work together.
